# Coagulation *factor V* gene 1691G>A polymorphism as an indicator for risk and prognosis of lower extremity deep venous thrombosis in Chinese Han population

**DOI:** 10.1097/MD.0000000000010885

**Published:** 2018-06-01

**Authors:** Chang-Lie Zhang, Zun-Min Li, Zhi-Hong Song, Tao Song

**Affiliations:** Department of Vascular Surgery, Linyi People's Hospital, Linyi, Shandong, China.

**Keywords:** 1691G>A, coagulation *factor V*, lower extremity deep vein thrombosis, polymorphism, prognosis, risk

## Abstract

The purpose of this study was to explore the negative influence coagulation *factor V* (*FV*) 1691G>A polymorphism had on the risk and prognosis of lower extremity deep venous thrombosis (LDVT) in Chinese Han population.

A total of 348 patients with LDVT (LDVT group) and 398 healthy individuals (control group) were selected to further this study. A polymerase chain reaction-restriction fragment length polymorphism method was used to analyze the *FV* gene 1691G>A polymorphism; coagulation and anticoagulation indexes of patients with LDVT were detected as a result. A 3-year follow-up and logistic regression analysis were conducted to determine the corresponding correlations between *FV* gene and LDVT.

In comparison with the control group, the frequencies of GA and AA genotypes and A allele of 1691G>A polymorphism significantly increased in the LDVT group. Also, in comparison with patients with LDVT carrying GG genotype of *FV* gene 1691G>A polymorphism, the following activities reduced significantly: prothrombin time, activated partial thromboplastin time, fibrinogen, protein C, and protein S, while activated protein C resistance and lupus anticoagulant positive rate increased in patients carrying A allele (GA + AA). Logistic regression analysis indicated that *FV* gene 1691G>A polymorphism, total cholesterol, low-density lipoprotein cholesterol, and LDVT family histories were all closely related with LDVT and were subsequent independent risk factors for LDVT. Moreover, patients with LDVT carrying A allele (GA + AA) had both higher patency and recurrence rates than those carrying GG genotype.

*FV* gene 1691G>A polymorphism may be associated with both the risk and prognosis of LDVT, potentially being a useful index for monitoring LDVT prognosis and risk.

## 1 Introduction

As one of the most common cardiovascular diseases, venous thromboembolism (VTE) affects about 2/1000 of the population and with possibilities of major morbidity and mortality rates.^[1]^ Deep vein thrombosis (DVT) forms a portion of the VTE and is also one of the most common cardiovascular diseases.^[2]^ Lower extremity DVT (LDVT) occurs due to an imbalance in pro-coagulation activity in coagulation homeostasis and DVT is a disease of high incidence, affecting approximately 1/1000 adults.^[3]^ Additionally, DVT has become a growing worldwide health concern with an annual incidence of 48 to 95 cases from Caucasian population of 100,000.^[4]^ LDVT is a multifactorial disease including both genetic and environmental risk factors such as old age, obesity, immobility, DVT history, drug abuse, recent surgery and acute infectious diseases. Strong genetic factors include genetic thrombophilia, antithrombin III deficiency and coagulation *factor V* (*FV*) Leiden mutation.^[5,6]^

The FV is a cofactor protein in coagulation and the *FV* gene is located on 1q24-25 consisting of 25 exons, resultantly transcribing a 6.8-kb mRNA.^[7]^ The pro-coagulant function of FV is primarily due to its interaction and cleavage by thrombin and active factor X, which is important as they are catalysts of blood clot formation.^[8]^ FV is linked to an increased risk of arteriovenous graft failure and in turn, anticoagulation treatment can reduce graft failure in patients with the GC or GG genotypes of *FV* gene.^[9]^*FV* mutation has been linked to osteonecrosis in Caucasian individuals as a possible genetic risk factor.^[10]^ The 1691 guanine-to-adenine substitution in *FV* gene (1691G>A) is a common gain-of-function polymorphism consisting of a prevalence of approximately 3% to 4% in the Italian population as well as being a congenital risk factor for VTE.^[11]^ Furthermore, a former study has shown evidence of the prevalence of *FV* polymorphisms (G1691A and A4070G) potentially increasing the risk of DVT occurrence, which provides additional evidence to support the idea that thrombophilic gene polymorphisms are linked to VTE.^[12]^ On the contrary, there are insufficient data focusing on the correlation between *FV* gene polymorphism and LDVT development. Our intentions involving this study is to investigate the role coagulation *FV* gene polymorphism plays in the risk and prognosis of LDVT in the Han population, thereby providing a useful index in monitoring LDVT prognosis and efficacy.

## 2 Materials and methods

### 2.1 Study subjects

Our study ran between May 2011 and January 2012 and during that time period, a total of 348 patients with LDVT (178 males, 170 females) of the Chinese Han population in Linyi People's Hospital were selected as part of the LDVT group. Inclusion criteria were as follows: all patients having received an angiography to detect DVT of the lower limb or echo-color-Doppler (ECD), confirming the presence of LDVT among the patients^[13]^; all patients between 23 and 82 years of age; all patients with complete and valid case data having received effective treatment for the first time and discharged from the hospital. Exclusion criteria include: patients who have had thrombopoiesis due to serious cardiovascular, liver, and kidney diseases, malignant tumors, fractures, recent surgery, acute infectious diseases, pregnancy, or other secondary factors. Secondary factors: patients receiving anticoagulant drugs within 1 week; and patients who have had a history of thrombosis in other parts of the body. Another 530 healthy individuals of Han population who received physical examination at Linyi People's Hospital in the same period were selected. Of the aforementioned 530, 398 cases without subclinical LDVT conditions confirmed by DVT of the lower limb or ECD (202 males and 196 females) were selected as the part of the control group. This study was approved by ethics committee of Linyi People's Hospital and signatures obtained through an informed consent by all participating patients and legal representatives.

### 2.2 Specimen collection

Fasting venous blood (10 mL) was collected from the patients with LDVT as well as healthy control subjects. Five milliliters of the collected blood was put into a heparin anticoagulant tube and centrifuged at a rate of 3000 rpm for 20 min. As we progress, the blood sample was stored in the Eppendorf tube separately to measure the total cholesterol (TC), triglyceride (TG), high-density lipoprotein cholesterol (HDL-C), low-density lipoprotein cholesterol (LDL-C), and both coagulation and anticoagulation indexes. The remaining 5 mL blood was kept in an ethylenediaminetetraacetic acid (EDTA)-contained tube and preserved in a −80°C cryogenic refrigerator for the extraction of genomic DNA.

### 2.3 Polymerase chain reaction-restriction fragment length polymorphism detection

The EDTA anticoagulant (0.2 mL) was subjected to genomic DNA extraction using a medium-amount blood genomic DNA extraction kit (Tiangen Biotech Co, Ltd, Beijing, China). Primer sequences were designed by Shanghai Sangon Bioengineering Co, Ltd (Shanghai, China). The forward primer was ATTGGTTCCAGCGAAAGC-3′ and reverse primer was CCATTATTTAGCCAGGAGACC-3′. Polymerase chain reaction (PCR) reaction system (20 μL) included 10 μL of 10× PCR buffer, 10 μL of deoxyribonucleoside triphosphate (dNTP), 0.4 μL of 25 mmol/L MgCl_2_, 1 μL each for upstream and downstream primers, 0.2 μL of 5 U/μL DNA polymerase (Beijing Biomed Biotechnology Co, Ltd, Beijing, China), 2 μL of DNA template, and 1.4 μL of high-performance liquid chromatography ultrapure water. The reaction conditions were as follows: predenaturation at 95°C for 3 min, followed by denaturation at 95°C for 30 s, annealing at 60°C for 30 s, and extension at 72°C for 1 min. A total of 30 cycles conducted with a concluding extension at 72 degrees for 5 min. The PCR product was examined by 2% agarose gel electrophoresis.

The PCR product was digested using *Mn1*I enzyme. The digestion system (20 μL) included: 10 μL of PCR product, 7 μL of DDH_2_O, 2 μL of enzyme digestion buffer, and 1 μL of *Mn1*I (Fermentas, Burlington, Canada). The reaction system underwent a water bath at 37°C for 16 h, examined by 2.5% agarose gel electrophoresis and photographed. The resulting genotypes were then identified and recorded.

### 2.4 Indexes detection

The TC, TG, HDL-C, and LDL-C concentrations were measured by an enzymatic assay and Hitachi 7600 automatic biochemical analyzer (Olympus Company, Tokyo, Japan). Lupus anticoagulant (LA) was detected by diluted Russell's viper venom time. The activities of protein C (PC), protein S (PS), and LA were detected by enzyme-linked immunosorbent assays. Activated protein C resistance (APCR) was detected using a coagulation assay. Coagulation parameters including prothrombin time (PT), activated partial thromboplastin time (APTT), fibrinogen (Fbg), and thrombin time (TT) were measured using a STA compact automatic coagulation analyzer (Stago Company, Chausson, French) and an original kit (STAGO Company). The coagulation analyzer had strict quality control requirements. All the values were measured based on quality control of that day.

### 2.5 Efficacy evaluation

All of the affected patients underwent low molecular weight heparin and urokinase for thrombolytic therapy. One week later, warfarin was ingested orally and the amount of warfarin was adjusted according to the patients and their coagulation function international normalized ratio (INR), and the warfarin was taken orally for approximately 6 to 12 months when the INR was stable at 2.0 to 3.0.^[14]^ Two weeks after treatment, venous ultrasonography and the assessment of patency rate were conducted in the affected limb of each patient both before treatment and 2 weeks after treatment and the latter was based on the standards raised by Porter and Moneta (numeric grading schemes for disease severity, risk factors, and outcome criteria of venous disease).^[15]^ The venous obstruction was assessed as follows: 0 point, each segment of the affected limb was completely unobstructed; 1 point, partially unobstructed; and 2 points, obstructed.

### 2.6 Follow-up

Once discharged from the hospital, patients were subject to subsequent check and follow-ups for an additional 3 years. The follow-ups concluded in December 2015. Symptoms and signs of each patient were recorded at each visit and angiography to detect DVT of the lower limb was conducted at the first, second, and third years or when patients had swelling, pain, or other symptoms. Recurrence is recorded only if thrombosis happened again in the already recovered venous segments. If patients had no swelling, pain, or other symptoms as well as deep venous venography showed complete patency of the affected limb, patients were instructed to stop taking warfarin.

### 2.7 Statistical analysis

All of the data were processed using the SPSS 21.0 software (SPSS Inc, Chicago, IL). The *t* test and χ^2^ test were performed to compare baseline characteristics between the LDVT group and the control group. Genotype distribution was tested using the Hardy–Weinberg equilibrium. Genotype distribution and allele frequency were analyzed using the *t* test. Logistic regression analysis was conducted to analyze the risk factors of LDVT. *P* < .05 was considered statistically variable.

## 3 Results

### 3.1 Baseline characteristics between the LDVT group and the control group

As shown in Table 1, there were 348 patients in the LDVT group, including 178 males and 170 females with a mean age of 55.9 ± 13.2 years. There were 398 patients in the control group, including 202 males and 196 females with a mean age of 56.8 ± 11.9 years. TC, TG, LDL-C, hormone therapy percentage, and LDVT family history percentage were higher in the LDVT group than those presented in the control group (all *P* < .05). There were no significant differences in age, gender, height, weight, hypertension, smoking history, a history of diabetes, or HDL-C between the LDVT and control groups (all *P* > .05).

**Table 1:**
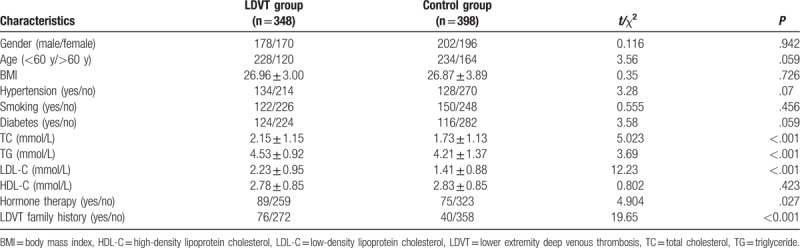
Baseline characteristics between the LDVT group and the control group.

### 3.2 PCR products of *FV* gene 1691G>A polymorphism

**Figure 1:**
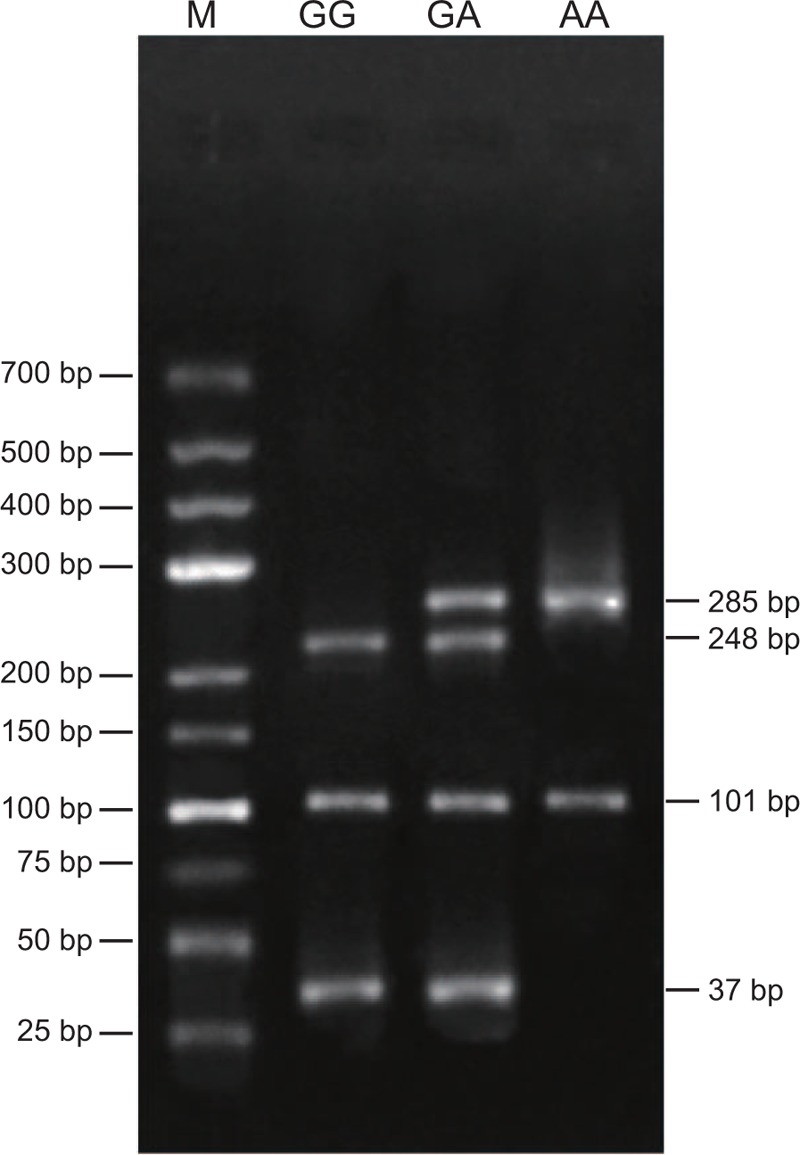
Electrophoresis results of digested fragments of each genotype of *factor V* 1691G>A polymorphism. 1 = GG genotype, 2 = GA genotype, 3 = AA genotype, M = Marker.

There were 2 restriction sites of *Mn1*I digestion in PCR amplification product. Three genotypes appeared following digestion: wild type (GG genotype) consisting of 3 fragments of 248, 101, and 37 bp; heterozygous mutation (GA genotype) consisting of 4 fragments of 37, 101, 248, and 285 bp; as for homozygous mutant (AA genotype), it contained the least amount of fragments consisting of 2 fragments of 285 and 101 bp (Fig. 1).

### 3.3 Genotype and allele frequency distribution of *FV* gene 1691G>A polymorphism

Goodness-of-fit χ^2^ test was used to evaluate the differences between observed and predicted frequency. According to this study, a total of 348 patients with LDVT were selected and the differences between the observed and predicted values of genotype and the allele frequencies of *FV* gene 1691G>A polymorphism were not significant (*P* > .05), indicating that the samples were obtained from a relatively large and randomly balanced population and were representative (Table 2).

**Table 2:**
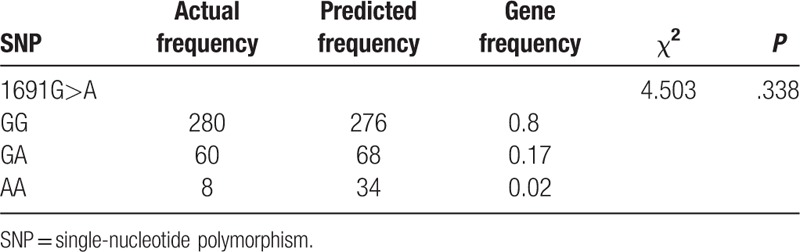
Hardy–Weinberg equilibrium testing of *factor V* gene 1691G>A polymorphism.

Significant differences were observed between the genotype and allele frequency distribution of *FV* gene 1691G>A polymorphism between the LDVT group and the control group (all *P* < .05). Compared with G allele, A allele might cause an increased risk of LDVT to 5.976 times (odds ratio = 5.976, 95% confidence interval [CI] = 3.450–10.350, *P* < .05). Compared with the GG genotype, there is a possibility that the AA and GA genotypes increase the risk of LDVT to 5.798 folds (95% CI = 3.292–10.210, *P* < .05) (Table 3).

**Table 3:**
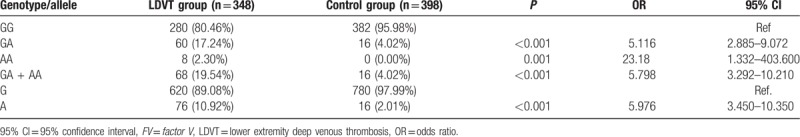
Genotype and allele frequency distribution of *FV* gene 1691G>A polymorphism in the LDVT group and the control group.

### 3.4 Comparison of coagulation and anticoagulation indexes in patients with different genotypes of *FV* gene 1691G>A polymorphism

In comparison with patients with LDVT carrying GG genotype of *FV* gene 1691G>A polymorphism, PT, APTT, Fbg, PC, and PS activities significantly reduced, while APCR and LA positive rates increased in patients carrying A allele (GA + AA) (all *P* < .05). On the contrary, there were no significant differences in TT between patients carrying the A allele and patients containing the GG genotype (*P* > .05) (Table 4).

**Table 4:**
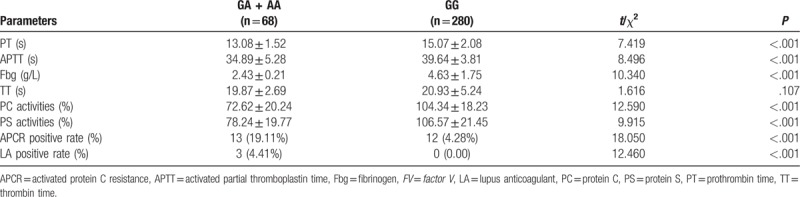
Coagulation parameters of patients with lower extremity deep venous thrombosis with different genotypes.

### 3.5 Logistic regression analysis of related factors for LDVT

The LDVT was used as a dependent variable. A logistic regression analysis suggested that the *FV* gene 1691G>A polymorphism, TC, LDL-C, and LDVT family histories were closely related with LDVT and were subsequently independent risk factors for LDVT (all *P* < .05). TG, smoking history, and hormone therapy were all found to be unrelated in regards to the risk of LDVT (all *P* > .05) (Table 5).

**Table 5:**
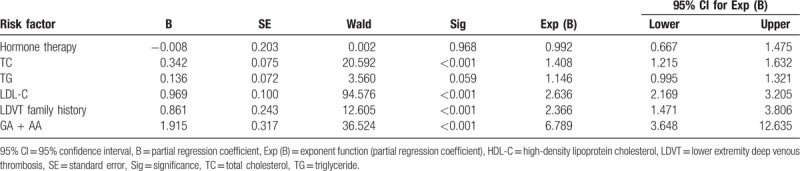
Logistic regression analysis of LDVT risk factors.

### 3.6 Correlation between different genotypes of *FV* gene 1691G>A polymorphism and prognosis of LDVT

During this follow-up, there were 14 patients not contacted, and the follow-up rate was 96.87%. Patency rates of the patients with LDVT before and after the treatment were recorded. patients with LDVT carrying the A allele (GA + AA) showed a higher patency rate than patients carrying the GG genotype (*P* < .05), indicating that A allele increased difficulty in the patients with LDVT and prolonged their time of treatment. The follow-up results showed that in comparison to the patients carrying the GG genotype, patients carrying the A allele (GA + AA) had a slightly higher probability of inducing other disorders (such as bleeding complications and post-thrombosis syndrome) but no significant genomic difference between them was found (all *P* > .05). Additionally, patients carrying A allele (GA + AA) displayed a significantly increased risk of LDVT recurrence compared to those patients carrying GG genotype (*P* < .05), thereby indicating that A allele was a key factor in LDVT formation and recurrence among the selected Han population (Table 6).

**Table 6:**
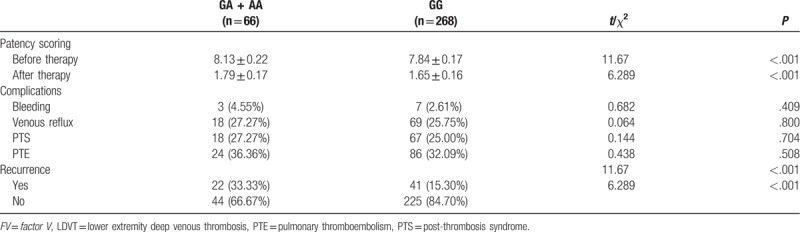
Correlation between *FV* gene 1691G>A polymorphism and efficacy of thrombolytic therapy and prognosis of patients with LDVT.

## 4 Discussion

The LDVT occurs due to an imbalance in pro-coagulation activity in coagulation homeostasis and DVT is a disease of high incidence. Our study aimed to investigate correlations between *FV* gene polymorphism and LDVT risk and prognosis in the selected Han population. This study showed that the frequencies between *FV* gene 1691G>A genotype and allele were significantly different among the LDVT and the control groups. In comparison with the GG genotype carriers of patients with LDVT, those who were carrying A allele had a 5.976 times more risk of LDVT. The heightened risk for LDVT indicated that *FV* gene polymorphism played an essential role in the development of this disorder. *FV* mutation involves a G-A substitution at nucleotide 1691, resulting in a pro-coagulant state by inducing FV resistance to inactivation by protein C, and *FV* G1691A mutation was one of the most common genetic risk factors for thromboembolism in Western countries.^[16]^ Moreover, the relationship between risk for VTE and *FV* gene polymorphisms may differ depending on different genotypes.^[11]^

Another result indicated that in comparison with the GG genotype carriers of patients with LDVT, PT, APTT, Fbg, PC, and PS activities remarkably reduced, while APCR and LA positive rates indicatively increased in patients carrying A allele of *FV* gene 1691G>A polymorphism. These results indicated that the role of *FV* gene polymorphism in LDVT might relate to the functions of *FV* gene in all these coagulation and anticoagulation indexes. PT, APTT, and Fbg concentrations are generally used for screening tests in the clinical setting for coagulation profile and reflect the activity of several coagulation factors found in both extrinsic and intrinsic systems.^[17,18]^ Prolonged APTT is a clinical indicator for the existence of coagulation inhibitors, with shortened APTT possibly reflecting increased levels of coagulation factors.^[19]^ Plasma Fbg level influences thrombogenesis, blood rheology, viscosity, and platelet aggregation. Fbg can also change to an aggregate of insoluble fibrin matrix after catalysis by thrombin.^[20]^ The PC pathway plays a very important role in the anticoagulant system and the corresponding congenital abnormalities of PC pathway are related with an increased risk of thromboembolic events (TEs).^[21]^ PS helps to downregulate thrombin formation and hereditary PS deficiency is a disorder that has been linked to an increased risk of DVT.^[22]^ APCR might have a relation to antiphospholipid antibodies which cause thrombotic events as evidenced by findings of more than 95% of APCR cases where due to the *FV* mutation, lead to a thrombotic event, primary venous thrombophilia.^[23]^ Further reports suggested that heterozygotic *FV* mutation and the presence of elevated LA levels were found in patients with an extensive unprompted DVT.^[24]^

Logistic regression analysis showed that *FV* gene 1691G>A polymorphism, TC, LDL-C, and LDVT family history were closely related with LDVT and were independent risk factors for LDVT diseases. Lipids may lead to the development of LDVT by regulating the fibrinolytic and coagulation systems required for its induction.^[25]^ Elevated TG level was linked with an increased risk of about double regarding VTE in postmenopausal women.^[26]^ Family history is a key essential risk factor for most unusual forms of VTE.^[27]^ LDL-C is a major controller of oxidative stress and endothelial dysfunction in cardiovascular disease, suggesting intensive LDL-C-lowering therapy by recent research.^[28]^ The *FV* gene 1691G>A variant was known as one of the most common genetic risk factors in the occurrence of VTE.^[29]^ The prevalence of *FV* Leiden mutation was elevated in patients with venous thromboembolic disease and those that carry *FV* Leiden seem to have direct correlation with the highest DVT development rates.^[30]^

Additionally, the study showed that patients with LDVT carrying A allele had a higher patency rate, slightly higher probability of inducing other disorders, and higher recurrence rates than patients carrying GG genotype, indicating that allele A increased treatment difficulty and time of patients with LDVT , proving to be a key factor of LDVT formation and recurrence in the Han population. As the most common cause of inherited thrombophilia, *FV* mutation was a significant risk factor in developing complications during pregnancy and additional adverse outcomes.^[31]^ Carriers of *FV* prothrombin mutation were at an increased risk of recurrent DVT after a first episode indicating their candidacy for anticoagulation.^[32]^

## 5 Conclusion

When administered simultaneously, coagulation and *FV* gene 1691G>A polymorphism were associated with an increased risk and poor prognosis of LDVT in the Han population by affecting coagulation and anticoagulation indexes. Besides, *FV* gene 1691G>A polymorphism, TC, HDL-C, and LDVT family histories were closely related with LDVT and were subsequently independent risk factors for LDVT. Our study could provide a potent genetic biomarker for the development of LDVT. To invoke better results and more precise estimates, more prospective studies should be conducted.

## Acknowledgment

The authors give their sincere appreciation to the reviewers for their helpful comments on this article.

## Author contributions

**Conceptualization:** Chang-Lie Zhang, Zun-Min Li, Zhi-Hong Song, Tao Song.

**Data curation:** Chang-Lie Zhang, Zun-Min Li, Zhi-Hong Song, Tao Song.

**Formal analysis:** Chang-Lie Zhang, Zun-Min Li, Zhi-Hong Song, Tao Song.

**Funding acquisition:** Chang-Lie Zhang, Zun-Min Li, Zhi-Hong Song, Tao Song.

**Investigation:** Chang-Lie Zhang, Zun-Min Li, Zhi-Hong Song, Tao Song.

**Methodology:** Chang-Lie Zhang, Zun-Min Li, Zhi-Hong Song, Tao Song.

**Project administration:** Chang-Lie Zhang, Zhi-Hong Song.

**Resources:** Chang-Lie Zhang, Zhi-Hong Song.

**Writing – original draft:** Chang-Lie Zhang, Zun-Min Li, Zhi-Hong Song, Tao Song.

**Writing – review & editing:** Chang-Lie Zhang, Zun-Min Li, Zhi-Hong Song, Tao Song.
